# Vagal Nerve Activity and Short-Term Clinical Outcomes after Stroke: What Is Left May Not Be Right

**DOI:** 10.3390/jcm12072446

**Published:** 2023-03-23

**Authors:** Samih Badarny, Amal Abu Ayash, Galina Keigler, Chen Hanna Ryder, Yori Gidron

**Affiliations:** 1 Department of Neurology, Galilee Medical Center, Nahariya 2210001, Israel; 2 Azrieli Faculty of Medicine, Bar Ilan University, Safed 1311502, Israel; 3 Brain & Behavior Research Institute, Western Galilee Academic College, Acre 2412101, Israel; 4 Department of Nursing, Faculty of Social Welfare and Health Sciences, Haifa University, Haifa 3498838, Israel

**Keywords:** stroke, stroke severity, hemispheric differences, vegal nerve activity, vegal nerve activity (HRV), stroke side

## Abstract

Stroke is a leading cause of death worldwide. Multiple factors influence the severity of stroke. Normal functional and biological differences seen between the hemispheres may also be related to stroke severity. In the present study, we examined the differences in the severity of stroke as a function of stroke side, and whether patients’ vagal nerve activity moderated such differences. We included 87 patients with an ischemic stroke, whose medical records were retrospectively examined for background information (age, gender), stroke side and severity by NIHSS, length of stay in hospital, inflammation such as C-reactive protein, and vagal nerve activity. The vagal activity was indexed by patients’ heart-rate variability (HRV), fluctuations in the intervals between normal heartbeats, derived from patients’ ECG. Results revealed that patients with left-side stroke had significantly worse NIHSS scores (10.6) than those with right-sided stroke (7.6, *p* < 0.05). However, when dividing the sample into those with low versus high HRV (at the median), only when HRV was low, did patients with left-side stroke have a worse NIHSS score (10.9) compared to those with right-sided stroke (6.5, *p* < 0.05). In contrast, no differences in stroke severity were seen between left stroke (10.2) and right stoke (8.7, *p* > 0.05), when HRV was high. These results tended to remain the same when statistically controlling for age effects, which was related to NIHSS, but not to the stroke side. These findings suggest that patients with left-sided stroke may have more severe strokes than those with right-sided ones, but that adequate vagal nerve activity may protect against such differences. Possible mechanisms and suggestions for future directions are provided.

## 1. Introduction

Stroke is a leading cause of morbidity and mortality worldwide, with up to 50% of survivors being chronically disabled [1]. This disability is a major aspect of having a stroke, and its type and extent depend on the location and amount of brain damage occurring due to a stroke. Among the long-term consequences of stroke are medical complications (e.g., seizures, urinary and bowel incontinence), musculoskeletal complications (spasticity, hypertonicity, hemiplegic shoulder or limb pain, wrist and hand flexions) and psychosocial complications (cognitive impairments, depression, anxiety, mood instability, and communication difficulties) [2]. Multiple risk factors for stroke have been identified including genetic, age, and gender differences, as well as lifestyle factors such as smoking and poor diet [3,4,5]. More recent stroke risk factors also include inflammation (excessive recruitment of innate immunity to “stressed” body regions), infection, pollution and cardiac atrial problems [6].

Another related but different issue is the search for factors determining stroke severity. Typically, stroke severity is assessed by the NIH stroke scale (NIHSS), which evaluates patients’ movements (eye, limbs, facial), sensation, linguistic abilities, balance, and attention. NIHSS is an index of stroke severity that presents the immediate level of disability, and it is an independent predictor of mortality following ischemic stroke [7]. Thus, NIHSS can serve both as an end-point on its own and as a prognostic factor. The NIHSS index is also correlated with levels and types of impairment in stroke patients [8]. This index is related to multiple functions including motor functions, consciousness, motoring functions, language and attention ability. Several patient factors correlate with a worse NIHSS score such as older age, lower hemoglobin, higher heart rate (HR), and higher aspartate aminotransferase [9].

One factor not fully investigated is the side of stroke and its relationship with stroke severity. This is of importance because the side of stroke is obviously related to different types of morbidity, depending on the location of brain lesions, which then affect certain brain and bodily functions. For example, lesions in the left cortex may affect linguistic performance while lesions to the right cortex could affect visuospatial abilities [10]. One study also found that left-sided strokes are more often clinically recognized and diagnosed than right-sided strokes, despite no differences in their prevalence using MRI scans [11]. This could happen since currently, the NIHSS score reflects to a greater extent disabilities which are affected more by the left hemisphere. However, is stroke side related to the severity of stroke, as assessed by the NIHSS index? The literature on cerebral asymmetry has also shown differences in immune modulation as a function of the hemispheres. The left hemisphere increases peripheral cellular and anti-viral immunity, while the right hemisphere reduces such immunity [12]. Given these neuro-immune differences between the two hemispheres, and given the different effects of each hemisphere on immunity, the effects of stroke side on stroke severity and prognosis require more research.

An additional factor which may protect against the severity of stroke on one hand, or affect the association between stroke side and stroke severity on the other hand, is the activity of the vagal nerve. This 10th cranial nerve is a major branch of the parasympathetic nervous system, it reduces blood pressure via the baroreflex, and high blood pressure is a known risk factor for strokes [13]. Vagal nerve activity is non-invasively indexed by heart rate variability (HRV), the fluctuations in the intervals between normal heartbeats [14]. HRV is a predictor of stroke incidence and post-stroke complications [15]. For example, in the longitudinal Copenhagen Holter study, which followed 676 stroke-free people, an inverse and strong relationship was found between the level of night-time HRV and risk of stroke [16]. Furthermore, the vagal nerve is an important neuroimmune modulator, as it reduces inflammation by two routes. First, the vagal nerve activates the hypothalamic–pituitary–adrenal axis, leading to the secretion of cortisol, which results in suppression of inflammation [17]. Second, descending vagal efferent branches convert at the celiac ganglion to sympathetic branches, which enter the spleen. In the spleen, these sympathetic branches bind to certain T-cells via beta-adrenergic receptors. These T-cells then secrete acetylcholine, which binds to its cholinergic receptor on splenic macrophages, to further inhibit the synthesis of inflammatory cytokines [18]. Yet, the role of the parasympathetic vagal nerve, indexed by HRV, in the relation between stroke-side and stroke severity, is unknown. Is stroke side related to stroke severity and does vagal activity play a role in this association? Vagal activity may also be affected by stroke side, because some studies showed that mostly the left hemisphere controls parasympathetic (vagal) activity [19]. Furthermore, some studies have found that HRV, the vagal index, interacts with other factors in relation to clinical outcomes. In one study, cancer stage predicted levels of tumor markers over time, but only when HRV was low [20]. In another study on patients with COVID-19, age predicted survival, only when HRV was low, but not when it was high [21]. The present study tested whether the side of stroke and HRV are related to stroke severity, indexed by the NIHSS level. In addition, we tested whether HRV moderates the relations between stroke side and other basic patient variables with NIHSS scores.

## 2. Methods

Design: This was a retrospective correlational study, conducted at the Galilee Medical Center-Nahariya, Israel, between January 2017 and December 2021.

Sample: Patients were included if they met the following inclusion criteria: Had an acute ischemic stroke, were within the age range of 18–90 years, were in the therapeutic window of ischemic stroke treatment, and received tPA. Since past studies included patients with and without tPA, and given its effects on clinical outcomes, we decided to include only patients with tPA treatment, in order to have a more homogeneous sample. Exclusion criteria included patients with hemorrhagic infarctions and having cardiac arrhythmias such as atrial flutter or atrial fibrillation. Of the 160 patients with acute ischemic stroke, 105 of them met the criteria for tPA treatment, but only 87 patients completed the treatment, because of uncontrolled hypertension, or nasal or mouth hemorrhage. The current study was approved by the Galilee Medical Center Helsinki ethics committee. Since the study is only using past existing data, there was no need for using informed consent forms.

### Measures

Background information: This included data on patients’ age, gender, and side of stroke.

Vagal nerve activity: This was indexed by patients’ heart-rate variability (HRV), based on patients’ 10sec ECG upon arrival at the hospital. Studies found that such ultra-short ECG HRV highly correlated with HRV derived from longer 5-min HRV measures [22]. Finally, multiple studies have shown that short and long measures of HRV predict prognosis in stroke [15]. We derived two HRV time domain parameters: The standard deviation of intervals between normal R-R intervals (SDNN) and the rout mean square of successive differences of adjacent R-R intervals (RMSSD).

Clinical outcomes: These included stroke severity by the NIHSS score, CRP at administration, and days of hospitalization. The NIHSS score considers patients’ movements (eye, limbs, face), sensation, linguistic abilities, balance, and attention. Its scores can range between 0 and 42, with scores greater than 25 reflecting a severe stroke. The NIHSS score also predicts mortality in stroke patients [7].

Statistical analysis: First, we present descriptive statistics, with means and standard deviations (SD) for continuous data and percentages for categorical data. Second, we examined associations between NIHSS scores and continuous data with Pearson correlations and between NIHSS and categorical data with t-tests. Finally, to examine the moderating role of HRV, we examined the relations between various study variables (age, stroke side) and NIHSS, after splitting the sample at the median HRV cut-off of the sample, and examining the associations separately in patients with low and high HRV.

## 3. Results

### 3.1. Descriptive Statistics

The sample included 33 women (37.9%) and 54 men (62.1%), 39 of them had a left-sided stroke (44.8%) and 48 had a right sided stroke (55.2%). In the full sample, patients’ mean and standard deviation (SD) age were 65.88 (12.38) years, mean (SD) CRP were 9.97 (16.08). Their mean (SD) disease severity score (NIHSS) was 8.94 (5.91) and their mean (SD) length of stay was 11.78 (14.41) days. Table 1 shows these data as a function of stroke side.

### 3.2. Relationship between Stroke Severity and Study Variables

Of all variables, NIHSS differed significantly as a function of stroke side. Patients with left-sided stroke had a worse NIHSS score (10.59) than those with right-sided stroke (7.60; t(64.47) = 2.32, *p* < 0.05). The gender distribution was not significantly different as a function of stroke side (X^2^(1) = 0.96, *p* > 0.05). In addition, age was positively and significantly correlated with NIHSS (r = 0.27, *p* < 0.05).

### 3.3. Does Vagal Activity Moderate Relations between Patient Variables and NIHSS Score?

We then examined whether the observed differences in stroke severity and patient varibles were moderated by patients’ vagal nerve activity, indexed by their SDNN. The sample was split at the median SDNN, namely 25 msec, to create equal group sizes as much as possible. The results were very similar using RMSSD, but we focused on SDNN as the effect appeared a bit stronger, and since more research has been conducted on the prognostic role of SDNN in cardiovascular diseases [23]. As shown in Table 2, only in patients with lower HRV, did those with left-sided stroke have significantly higher levels of NIHSS (10.95) than those with right-sided stroke (6.46; t(32.79) = 2.35, *p* < 0.05). These differences were absent in patients with higher HRV. In addition, only in patients with lower HRV, did those with left-sided stroke have significantly lower levels of log-CRP (0.58) than those with right sided stroke (0.86; t(43) = 2.02, *p* = 0.05). Again, these differences were absent in patients with higher HRV (*p* > 0.05). Finally, only in patients with low HRV, did age significantly and positively correlate with NIHSS (r = 0.39, *p* = 0.007), while age was unrelated to NIHSS in patients with high HRV (r = 0.11, *p* > 0.05).

## 4. Discussion

This study examined whether patients with different stroke sides differ in indexes of stroke severity and LOS. First, we found that patients with left-sided stroke had more severe strokes than those with right-sided stroke, as indexed by their NIHSS score. Second, we found that several differences between right- and left-sided stroke were moderated by patients’ vagal nerve activity, indexed by their HRV. Specifically, only in patients with low HRV, did those with left-sided stroke have a worse NIHSS score than those with a right-sided stroke, while this difference in NIHSS did not occur in patients with high HRV. In addition, only in patients with low HRV, did age correlate significantly and positively with NIHSS, but not in patients with high HRV. Finally, and again, only when HRV was low, patients with right-sided stroke had significantly higher inflammation, indexed by CRP, than those with left-sided stroke.

How can we explain this pattern of results? Few if any studies had examined differences between left- and right-sided stroke and stroke severity, as indexed by the NIHSS score. As mentioned above, left-sided stroke is more often apparent and diagnosable than right-sided stroke [11]. Furthermore, our results are in line with a large-scale study, which also found that patients with left-sided stroke had worse NIHSS scores than those with right-sided stroke [24]. First, two of the items on the NIHSS scale refer to language/speech, which is usually controlled more by the left hemisphere, including speech comprehension in different condition [25] and especially speech production [26]. Thus, it is possible that stroke-related cortical damage on the left side may have contributed more to language deficits, leading to worse NIHSS scores, compared to patients with right-sided stroke. However, we did not examine the scores on each item separately and this needs to be examined in future studies. Second, we also found that high HRV moderated or protected against age effects and against the effects of stroke side on stroke severity. Yet, this protective role of HRV in the side-NIHSS relation needs to be taken with caution because, in patients with high HRV, both those with left- and right-sided stroke had relatively high NIHSS scores. Only when HRV was low did patients with left-sided stroke have worse NIHSS scores than those with right-sided stroke. Nevertheless, the moderating role of the vagal nerve in the effects of other disease-related factors on clinical outcomes has been found in other diseases such as cancer. In one study, a worse tumor stage predicted higher tumor marker levels later, in both prostate and colon cancer, but only when HRV was low, not when HRV was high [27]. In a study on the relations between life events and cancer risk, life events predicted risk of cancer only in people with initially low, but not high HRV [28]. In the present study, high HRV emerged to protect against the possible effects of stroke side and age on stroke severity.

Is there evidence directly showing vagal nerve neuroprotection at all and in stroke specifically? Some studies suggest that electric vagal nerve stimulation increases recovery following spinal cord injury, by reducing neuroinflammation via upregulating expression of the alpha-7 nicotinic acetylcholine receptor [29]. This is a crucial step in the cholinergic vagal anti-inflammatory reflex [18], because by binding to that receptor, macrophages then synthesize less pro-inflammatory cytokines. Furthermore, a study conducted in rats induced to have ischemic strokes showed that vagal nerve stimulation paired with “rehabilitation training” led to greater limb recovery compared to “rehabilitation training” alone [30]. Such neuroprotective effects may explain why in patients with high HRV, no differences in NIHSS were found between those with left versus right stroke, and these effects may also explain why age did not correlate with NIHSS in patients with high HRV. The neuroprotective effects of the vagus in stroke need further investigation.

There is also evidence linking vagal activity and specifically HRV with the left hemisphere. In one study, people exposed to repetitive transcranial magnetic stimulation (rTMS) on the left dorsolateral pre-frontal cortex showed higher levels of HRV than controls [31]. In an earlier study, parasympathetic changes were found when viewing an emotional film only when it was exposed visually to the left hemisphere [19]. Though the brain laterality of HRV is not fully understood, it is possible that a left-sided stroke may reduce the neuroprotective effects of the vagus, possibly resulting in a worse stroke. Furthermore, such a worse NIHSS score may be manifested even more in patients who initially have low HRV, as observed in the present study. However, this explanation must be taken with caution because we did not observe differences in HRV between patients with left- versus right-sided stroke. Perhaps the combination of a left-sided stroke and low HRV may result in a worse stroke, as found in this study. The findings in the present study clearly suggest such a synergistic effect.

The finding that only when HRV was low, patients with right-sided stroke had significantly higher inflammation (CRP), than those with left-sided stroke, may be understood by the hemispheric differences in immunity. In a review of 11 studies, the left hemisphere was shown to increase anti-viral immunity while the right hemisphere reduces such immunity [12]. When there are lesions in a hemisphere, as in seizure surgery, these patterns reverse [32]. However, the differential effects of each hemisphere on peripheral inflammatory markers have received little research attention. Based on converging evidence, it is possible that stroke-induced neuronal loss in the right side, the side that also mediates more sympathetic activity, which in some cases increases inflammation [33], may result in more peripheral inflammation particularly in people with lower HRV, reflecting absence of vagal anti-inflammatory modulation [18]. This pattern of hemispheric differences, stroke side and peripheral inflammation, requires further research with larger samples.

The present study included several limitations. First, the sample size was not large. This hampered a comparison of specific NIHSS items in patients with right- versus left-sided strokes, to reveal which aspects of stroke severity differ as a function of stroke side. Second, this was a retrospective study, not enabling us to control how the ECG was performed, from which HRV was derived. Future studies need to perform larger studies with a longitudinal design. Such a study could examine whether the stroke side and initial HRV predict stroke severity and prognosis over time. Third, we excluded patients with hemorrhagic stroke. These observations also need to be tested in patients with hemorrhagic stroke. Finally, we did not examine certain complications (e.g., pneumonia, thrombosis) and their effects. Future studies should examine their prevalence in left- and right-sided strokes and their differential effects on NIHSS, as functions of stroke side and HRV levels.

Nevertheless, this study showed that stroke side and age may matter in relation to stroke severity and that such differences may be augmented by reduced vagal nerve activity (HRV). Today, there are numerous ways to measure HRV including with simple devices placed on patients’ left index finger, using photo-plethysmography technology. Such technologies were found to predict prognosis in various diseases such as inflammatory bowel disease [34]. The clinical implications of our observation are that due to the ease of measuring HRV in patients, clinicians could use HRV to estimate patients’ vulnerability to a worse stroke related either to stroke side or to their age. Knowing the HRV in advance from past clinical visits could even help to identify patients vulnerable for dying.

Since it is possible to activate the vagus via HRV-biofeedback with slow-paced breathing or electorally via non-invasive stimulators, the effects of such vagal nerve activation on the severity of stroke in patients with left-sided stroke need to be examined in future randomized controlled trials [35]. From a scientific point of view, these observations call for considering vagal activity as an important factor which may determine relations between certain clinical variables and stroke severity. Furthermore, we found that vagal activity may moderate the influence of known prognostic factors such as age and inflammation. This calls for an integrative system-approach where we consider the nervous system (vagal nerve), inflammation and hemispheric factors in stroke outcomes.

## Figures and Tables

**Table 1:** Means (SD) for main study variables as function of stroke side.

Variable	n = 39		n = 48	
	Left-Stroke		Right-Stroke	
	Mean	SD	Mean	SD
Age	68.33	(12.91)	63.90	(11.69) !
NIHSS	10.59	(6.86)	7.60	(4.66) *
Log-CRP	0.64	(0.49)	0.78	(0.42)
Log-length of stay	0.90	(0.48)	0.76	(0.44)
Log-SDNN	1.43	(0.35)	1.51	(0.43)

Note: SD = standard deviation; ! *p* < 0.10; * *p* < 0.05.

**Table 2:** Means (SD) for main study variables as function of stroke side and level of vagal activity (HRV).

Variable	Low HRV				High HRV			
	Left-Stroke		Right-Stroke		Left-Stroke		Right-Stroke	
	Mean	SD	Mean	SD	Mean	SD	Mean	SD
NIHSS	10.90	7.47	6.46	4.69 *	10.22	6.27	8.75	4.43
Log-CRP	0.58	0.54	0.86	0.39 *	0.72	0.42	0.70	0.44
Log-length of stay	0.84	0.48	0.63	0.44	0.98	0.48	0.89	0.42

Note: HRV = heart rate variability; NIHSS = NIH severity score; CRP = C-reactive protein; * *p* ≤ 0.05.

## Data Availability

The data used in this study cannot be made publicly available due to privacy and ethical concerns as per the guidelines set forth by the Helsinki ethics committee. Access to the data is restricted by the Galilee Medical Center Research Authority.

## Abbreviations

SDNN	Standard Deviation of NN intervals
RMSSD	Root Mean Square of Successive Differences between normal heartbeats
NIHSS	National Institutes of Health Stroke Scale
CRP	C-Reactive Protein
HRV	Heart-Rate Variability
rTMS	repetitive Transcranial Magnetic Stimulation
HR	Heart Rate
LOS	Long of Stay
